# Sustainable financing of AYSRH programs by local governments through the TCI model

**DOI:** 10.3389/fgwh.2023.1060857

**Published:** 2023-03-30

**Authors:** Levis Otondi, Nancy Aloo, Peter Kagwe, Assumpta Matekwa, Kenneth Miriti, Lilly Njoki, Denis Joel Sama, Kenneth Owino, Paul Nyachae

**Affiliations:** ^1^ The Challenge Initiative Project, Jhpiego Kenya Country Office, Nairobi, Kenya; ^2^ Department of Health, County Government of Kilifi, Kilifi, Kenya; ^3^ Department of Health, County Government of Migori, Migori, Kenya; ^4^ The Challenge Initiative Project, Jhpiego Uganda Country Office, Kampala, Uganda

**Keywords:** adolescent, sexual, reproductive, health, program, financing, sustainable, local government

## Abstract

**Introduction:**

Despite the existence of a legal policy framework, financing of adolescent and youth sexual and reproductive health (AYSRH) services has remained weak. External donors are the main financing agents, which has implications for the sustainability of service provision. International development partners have reduced funding for health programs from historically high levels. In Kenya, the health sector's budget allocation has remained below the 15% committed to under the Abuja declaration. With Kenya's devolved government structure, a greater proportion of financial resources are dedicated towards recurrent and structural expenses as opposed to addressing health systems gaps.

**Objectives:**

The purpose of this manuscript is to assess the contribution of The Challenge Initiative (TCI) Business Unusual model on AYSRH services in the counties of Kilifi and Migori, as well as to examine the institutionalization of high impact interventions (HIIs) within the annual work plan, budget, and systems of the said counties. Additionally, this study aims to analyse the trend in contraceptive uptake among adolescent and young women aged 15 to 24 in Kilifi and Migori counties.

**Methods:**

Migori and Kilifi Counties chose to partner with TCI to implement the Business Unusual model. Interested counties apply for the initiative's support and commit to contributing a portion of the funding needed to adapt and implement high impact interventions (HIIs). Based on the identified gaps, TCI supported the counties to prioritize the HIIs including integrated outreaches, youth fixed days, whole site orientation, youth champions, and youth dialogues. The program was implemented between July 2018 to June 2021 in 60 and 68 public health facilities of Kilifi and Migori Counties, respectively. The county teams identified and selected program implementation team whose key role was to coordinate, review, monitor, mobilize resources and report AYSRH program implementation progress.

**Results:**

The results showed a 60% increase in financial commitments on AYSRH programming from 2018 to 2021 in both counties. The average expenditure for committed funds for Kilifi and Migori Counties was 116% and 41% respectively. As the counties continued to allocate and spend funds on the implementation of HIIs, there was a noticeable increase in contraceptive uptake among the young people aged 15 to 24 who visited health facilities for services. There was a 59% and 28% percentage increase in contraceptive uptake among young people (15–24 years) between 2018 and 2021. The proportion of adolescents amongst those presenting for first ANC clinic dropped from 29.4% in 2017 to 9% in 2021 in Kilifi County and from 32.2% in 2017 to 14% in 2021 in Migori County. Using the TCI's *Sisi kwa Sisi* coaching model of lead-assist-observe-monitor, 20 master coaches were trained. The master coaches cascaded the training to over 97 coaches. The coaches will continue to build capacity of peers in advocacy for resource mobilization and implementation of HIIs. At least nine of TCI's HIIs have been adopted in Kilifi and Migori County strategies and annual work plans, and there is financial support for their sustainability.

**Discussion:**

The increase in adolescent contraceptive uptake might have been as a result of the system strengthening through self-financing of AYSRH programs, the institutionalization of HIIs, and the coaching. Local governments can invest in and sustain their own AYSRH programs, which will lead to an improvement in adolescent and youth access to contraceptive services and, as a result, a reduction in adolescent pregnancies, maternal mortality, and infant mortality.

## Introduction

In the recent past, adolescent youth sexual reproductive health (AYSRH) needs have become a growing concern worldwide. The high number of adolescent pregnancies is attributed to inadequate access to sexual reproductive health services. This has exacerbated the vulnerability of adolescent young girls to poverty, exclusion, and exploitation (1). Adolescent pregnancy is a major health and social concern due to its association with high maternal and child morbidity and mortality (1, 2). In Kenya, 47% of young women aged 18–24 years reported sexual debut before the age of 18 years, with a teen pregnancy rate of 18% (3).

Kilifi and Migori counties are among the counties recording high rates of adolescent pregnancies in Kenya. In 2018, the proportion of adolescent pregnancy (10–19 years) among women attending first antenatal clinic (ANC) in Kilifi and Migori stood at 29.4% and 32.2% respectively, much higher than national level (4).

The high adolescent pregnancy is attributed to low contraceptive utilization among sexually active adolescents. According to Saifuddin et.al 2019, family planning programs have contributed to an increase in the contraceptive prevalence rate globally (5). Increasing use of contraceptive methods will not only result in improvements in health-related outcomes such as reduced adolescent pregnancy, reduced maternal and infant mortality, but creates an opportunity for women and girls to advance in their education and careers while improving their economic outcomes (6, 7).

During the 2019 International Conference on Population and Development (ICPD), governments agreed to enhance access to comprehensive AYSRH services and responsive information and education for all adolescents and youth (8). This will enable them to make informed decisions and choices about their sexuality and reproductive lives, to adequately protect themselves from unintended pregnancies.

Resource allocation and utilization towards AYSRH interventions enhances program improvement and sustainability. Kenya is a signatory of the Abuja Declaration, which pledges to increase African governments' spending for health to at least 15% of the total budget (9), but this commitment remains to be met (10). According to Korir et al., international NGOs and foundations have been the main financing agents in the provision of family planning (FP) services in the country and it is not sustainable (11). Continuous advocacy efforts play a key role towards funds allocation and utilization for sustained implementation of AYSRH interventions. According to Starrs et al. 2018, to sustain the gains in AYSRH service provision and reduction in adolescent pregnancies, health systems need sustainable and predictable financing. County governments must make essential sexual and reproductive health services a priority and ensure they are included in health budgets. Provision of resources towards AYSRH is critical to ensure increased uptake of contraceptives among adolescents and reduction of adolescent pregnancies (12).

Although donor assistance for AYSRH Programs in Kenya has increased over the years, the percentage of government funds devoted to AYSRH program is still low (13), and Kilifi and Migori counties are not exceptional. With devolved government structures in Kenya, counties have assumed a greater role and responsibility including allocation and expenditure of funds in the implementation of AYSRH programming. Nonetheless, a greater proportion of the financial resources are usually dedicated towards human resources, operations, and maintenance as opposed to addressing health systems and service delivery gaps (14).

The Challenge Initiative (TCI) has been working in collaboration with the county Government and other implementing partners in Kilifi and Migori counties to implement select global best practices in AYSRH under the Business Unusual Model. The Business Unusual Model helps in prioritization of finance, scale-up, and sustainability of high-impact solutions for family planning and AYSRH. The select best practices are referred to as high impact interventions (HIIs). This approach is a shift in strategy from the traditional development model, in that it is demand-driven, allowing counties to self-select, as well as determine FP and AYSRH HIIs to implement based on existing gaps. This concept has cultivated a mind-set shift towards resource allocation, increased ownership, and sustainability of AYSRH of programs. The TCI program is currently being implemented by city governments in 11 countries in Africa and Asia.

This paper aims to share experiences on how the TCI Business Unusual Model has contributed to the success of the AYSRH program in Kilifi and Migori counties leading to increased uptake of contraceptives and reduced adolescent pregnancies. Specifically, we present the contribution of TCI Business Unusual Model on the improvement of AYSRH program sustainability; and document trends in contraceptive uptake among adolescent and young women aged 15- 24; and adolescent pregnancy among 15–19-year-olds in Kilifi and Migori during the implementation period.

## Methods

### Program description

In 2010, the Bill & Melinda Gates Foundation launched the Urban Reproductive Health Initiative (URHI) to identify solutions to address the FP needs of the urban poor. The URHI demonstrated the effectiveness of a range of supply- and demand-side interventions in improving the accessibility, quality, and use of FP services. To catalyse the rapid adoption and scale-up of these high-impact interventions (HIIs), the foundation created TCI. Launched in 2017, TCI deploys a “business unusual” model, whereby interested local governments apply for the initiative's support and commit to contributing a portion of the funding needed to adapt and implement HIIs in their geographies. A diverse range of HIIs available to local governments include interventions to improve management of FP programming, support advocacy to increase funding for FP, improve service delivery, and generate demand. Starting in 2018, local governments could also elect to “layer” on programming to support adolescent and youth sexual and reproductive health (AYSRH). To effectively implement these interventions, local governments received a range of support, collectively called TCI-University. These include:

• Toolkits with detailed protocols, job aids and manuals to guide implementation of the HIIs;
• Coaching to build the managerial and technical capacity of local government officials, service providers, community health workers (CHWs), and other community actors;
• Access to a community of practice (CoP) to share learnings and experiences.

### Study setting

Migori and Kilifi Counties, located in Kenya, are among the counties that chose to partner with TCI to implement the Business Unusual model. TCI supported the counties to prioritize the HIIs based on their existing gaps. The selected HIIs included; integrated outreaches, youth fixed days, whole site orientation, youth champions, intergenerational youth dialogues and digital/media platforms. The program was implemented between July 2018 to June 2021 in 60 and 68 public health facilities of Kilifi and Migori counties, respectively. The county teams identified and selected key program implementers, referred to as program implementation team (PIT) whose key role was to coordinate, review, monitor and report AYSRH program implementation progress. The PIT also played a role in advocacy for resource mobilization, allocation, and utilization for AYSRH Programming.

### Indicators

The various indicators of program progress were regularly monitored using data from the following information systems:

• Kenya Health Information System (KHIS): used to track contraceptive uptake service data among adolescents aged 15–19 and 20–24 years from all health facilities reporting into KHIS. This system also captured antenatal care (ANC) attendance from those presenting with pregnancy at first ANC visit at facility aged 15–19 years in Kilifi and Migori counties. This data was used to calculate the total number of adolescents pregnant in a given financial year.
• Program progress reports: these tracked program outputs, financial expenditure and system strengthening qualitative data. Financial data was reviewed from existing documents like annual work plans and financial commitments made by Kilifi and Migori counties for the financial year 2017 to 2021. Financial expenditure on AYSRH was tracked and reviewed against the county budgets, compared before and after intervention.
• Most Significant Change (MSC) technique was used to capture voices of the implementers, stakeholders and community members about the impacts of the AYSRH program on their lives and communities. MSC stories were also collected from targeted key decision makers at the county level and healthcare providers.

## Results

### Funds allocation and expenditure

Over the years of program implementation there were increases in financial commitments and expenditure on AYSRH Program in both Kilifi and Migori Counties. Kilifi County spent more than they had initially committed which demonstrates the County's commitment to addressing adolescent health matters (see Table 1 below).

**Table 1:** Financial commitment and expenditure on AYSRH.

Period	FY 2018/2019		FY 2019/2020		FY 2020/2021	
Finances (AYSRH)	Kilifi	Migori	Kilifi	Migori	Kilifi	Migori
Geography commitment (USD)	15,000	20,000	71,760	30,000	101,200	60,000
Geography Expenditure (%)	15,150 (101%)	6,800 (34%)	91,135 (127%)	12,900 (43%)	121,548 (120%)	28,070 (47%)

Source: TCI program management information system (PMIS).

### Health systems strengthening

#### Capacity transfer

TCI trained 20 master coaches who cascaded the training to over 97 coaches in Kilifi and Migori to ensure provision and implementation of AYSRH HIIs. The five-day standardized training curriculum broadly entailed preparing master coaches at county level to spearhead the implementation of the AYSRH program, applying transformative leadership and coordination to implement the AYSRH program, how to adapt and implement HIIs using the TCI—University toolkit, managing resources effectively and utilizing data for AYSRH program monitoring and decision making.

Some of the interventions targeting adolescents implemented included 700 AYSRH whole site orientation sessions—an approach to provide basic AYSRH knowledge to all staff (clinical & non-clinical) working in the health facility, 209 integrated youth outreaches—a health service delivery activity done outside the health facility aimed at bringing services closer to the youths, 184 youth dialogue days—a dialogue forum that bring young people together to discuss their needs and challenges, training 145 youth champions—young people who advocate for AYSRH services- trained on AYSRH, and 15 advocacy meetings in both Kilifi and Migori supporting increased expenditure and commitments for the next financial years.

#### Institutionalization of the HIIs

The MSC stories collected demonstrated that HIIs and processes, including the establishment of AYSRH coordinators, have been institutionalized in Kilifi and Migori County health systems, budgets and annual work plans, and there is support for their scalability. Here are quotes from select key county leadership and health management teams.

“*The TCI’s business model came in at the right time, and as a county, we had to adopt and adapt the interventions to start the journey in reducing our negative indicators in AY. (Adolescent and Youth)*”

Political leader- Kilifi County.

“*When TCI introduced the adolescent and youth program we knew we had to be part of it…. We did not even have a policy as a county to guide us on how to implement AY programs. One of the indicators to be addressed was contraceptive uptake among adolescents……, we focused on interventions that would also reach the youth. We had a starting point in that we picked interventions that had worked*.”

County Health Department representative—Kilifi County.

“*The establishment of the County AYSRH Coordinators’ position ensured that the program gets a differentiated share of budget allocation from the main Reproductive Maternal Newborn Child and Adolescent Health (RMNCAH) budget, it was given space in the annual work plan as a subprogram within RMNCAH. The Co-ordinator was tasked with consolidating all the partners together hence establishment of AYSRH Technical Working Group (TWG). The co-ordination mechanism also saw the development of the county AYSRH strategic plan document, which borrowed a lot from TCI-University, to guide AYSRH programming*.”

County Adolescent Health Representative—Kilifi County

“*The position of county AY coordinator enabled the mapping of all AY stakeholders and formation of taskforce including the development of Migori county AY multisectoral action plan which borrowed a lot of HIIs from the TCI—University. The positions were also cascaded down to all the 8 subcounties*”

County Adolescent Health Representative—Migori County

### Adolescent visits to health facilities for services

In both Kilifi and Migori Counties, there was an increase in contraceptive uptake among adolescents (15–24 years) with a percentage increment of 59% and 28% respectively over the period of three years. However, there was a decrease of 21% in from 2018/2019 to 2019/2020 in Kilifi County for the 15–19 years (see Table 2 below). The start of Coronavirus Disease 2019 (Covid-19) pandemic and its related restrictions could have led to the decrease in the uptake of contraceptives.

**Table 2:** Adolescent 15–24 years visits to health facilities for contraceptive services (Total number of adolescent visits to health facilities, percentage increase of adolescent visiting for contraceptive services for the period year).

Period (FY)	15–19		20–24	
	Kilifi	Migori	Kilifi	Migori
2017/2018	9,106	21,841	28,862	35,126
2018/2019	17,062 (87%)	25,120 (15%)	46,816 (62%)	41,900 (19%)
2019/2020	13,431 (-21%)	27,715 (10%)	46,990 (0.37%)	45,561 (8.7%)

Source: KHIS—MOH 711.

## Study limitations

The lack of age-disaggregated facility data on adolescents presenting with pregnancy and the absence of surveys hindered impact assessment on contraceptive uptake by age and measures of universal contraceptive services coverage. During the study period, data on adolescent contraceptive access from private facilities, including pharmacies and drug shops, was not obtained.

## Discussion

Since 2018, both Kilifi and Migori Counties of Kenya progressively increased their financial commitments to AYSRH Program. This indicates that these counties have appreciated the importance of addressing the prevailing sexual and reproductive health related challenges which exacerbate the vulnerability of adolescents and youth to poverty, exclusion, and exploitation. These counties have assumed a greater role and responsibility of abating AYSRH challenges within their jurisdiction. This progress makes Kilifi and Migori Counties particularly exceptional in a country where the percentage of government funds devoted to AYSRH program is small despite increasing donor assistance for AYSRH Programs. The increase in financial commitments towards AYSRH by Kilifi and Migori Counties is in accordance with the treaties of the 2019 ICPD, where governments, including Kenya, agreed to enhance access to comprehensive AYSRH services and responsive information, education to all adolescents and youth (8). This development in the two counties also follows the Abuja Declaration (9).

The sustained increased allocation of more resources to tackle AYSRH challenges within these counties may have resulted from successful smart advocacy efforts by various partners, including TCI, which cultivated a mind-set shift towards resource allocation, increased ownership, increased adolescent contraceptive uptake and sustainability of AYSRH of programs. According to Korir et al., international NGOs and foundations have been the main financing agents in the provision of FP services in the country and it is not sustainable (11). If Kilifi and Migori continue with the same trend of financial commitment and allocation to AYSRH, then we may see sustained programs in these two counties in future. According to Starrs et al. 2018, to sustain the gains in AYSRH service provision and reduction in adolescent pregnancies, health systems need sustainable and predictable financing. County governments must make essential sexual and reproductive health services a priority and ensure they are included in health budgets. Provision of resources towards AYSRH is critical to ensure increased uptake of contraceptives among adolescents and reduction of adolescent pregnancies. Continuous advocacy efforts play a key role towards funds allocation and utilization for sustained implementation of AYSRH interventions.

The relative increase in expenditure on AYSRH Programs in both Kilifi and Migori Counties illustrate enthusiasm of the counties' structures to implement planned AYSRH interventions, which could eventually abate the prevailing AYSRH challenges, especially high rates of adolescent pregnancies. Evidence from research studies and other projects shows that effective FP interventions improve adolescent access to contraceptive services and hence reduction of adolescent pregnancies, maternal and infant mortality.

**Figure 1:**
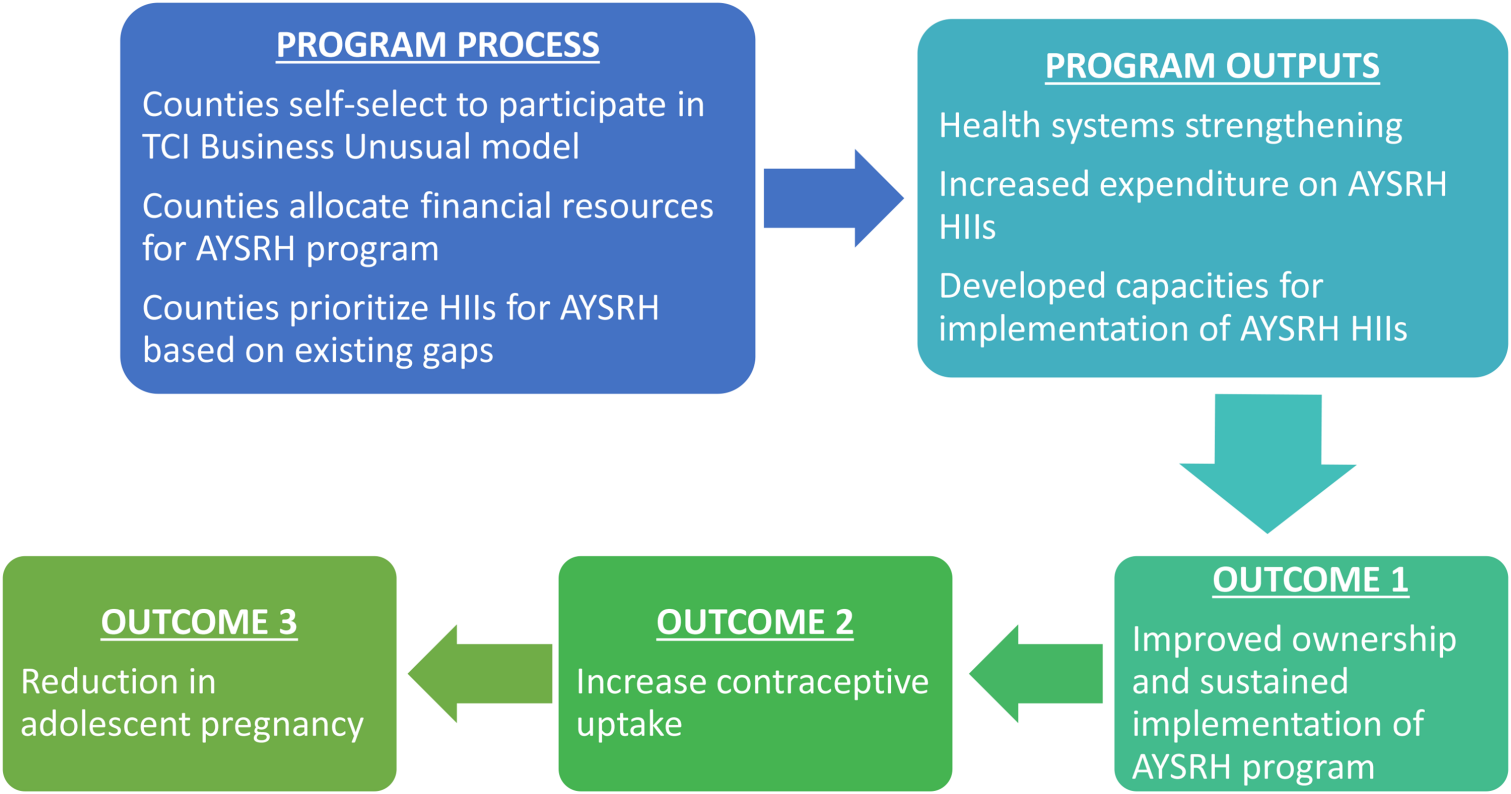
Conceptual framework.

The allocated funds were spent in implementation of AYSRH Program HIIs. These HIIs have been adapted into Kilifi and Migori Counties' strategies and annual work plans, which increases the likelihood of sustained implementation. The early positive and promising results from the interventions may inspire the leadership of these two counties to sustain allocation of funds for AYSRH Program in future. The establishment of the position of AYSRH Coordinators across the two counties would strengthen coordination, accountability, management, and implementation quality of the AYSRH HIIs and ensure use of evidence for action. The conceptual framework, depicted in Figure 1, outlines the processes, outputs, and expected outcomes of the TCI program to ensure sustainable financing for AYSRH programs. The framework can be adapted by other implementers to achieve the same goals.

## Conclusions and recommendations

Local governments can invest and sustain their own AYSRH programs contributing to improved adolescent and youth access to contraceptive services and hence reduction of adolescent pregnancies, maternal and infant mortality. This would also create an opportunity for women and girls to advance in their education and careers while improving their economic outcomes. To meet the needs and fulfill the rights of adolescents and youth, local governments should allocate funds and ensure their utilization for AYSRH interventions. Galvanization of political will and commitment for AYSRH at county leadership will ensure prioritization of adolescent health interventions. Continuous advocacy will ensure allocation and release of funds for health programs by empowering local community-based organizations, civil society organizations and youth groups to hold the leaders accountable on their promises. Local governments should embrace public private partnerships to leverage on resources in expanding FP commodities and services availability.

## Data Availability

The original contributions presented in the study are included in the article/Supplementary Material, further inquiries can be directed to the corresponding author/s.
